# *In silico* analysis of a major allergen from *Rattus norvegicus*, Rat n 1, and cross-reactivity with domestic pets

**DOI:** 10.12688/f1000research.20534.2

**Published:** 2019-12-12

**Authors:** Marlon Munera, Neyder Contreras, Andres Sánchez, Jorge Sánchez, Yuliana Emiliani

**Affiliations:** 1 Medical Research Group (GINUMED) Universitary Corporation Rafael Nuñez, Cartagena, Colombia; 2 Group of Clinical and Experimental Allergy (GACE), IPS Universitaria, University of Antioquia, Medellin, Colombia

**Keywords:** Allergen, lipocalin, cross-reactivity, docking, in silico

## Abstract

**Background: **Lipocalins play a role in the cellular trafficking of pheromones and are involved in allergic responses to domestic pets. However, the cross-reactivity among allergens of this group has been poorly explored, and the pheromone linking capacity is not well characterized. The aim of this study was to explore cross-reactive epitopes and pheromone linking capacity among Rat n 1 and homologues in domestic pets through an *in silico* approach.

**Methods: **ElliPro and BepiPred *in silico* tools were used to predict B cell linear and cross-reactive epitopes. The pheromone linking capacity was explored by docking virtual screening with 2-ethylhexanol, 2,5-dimethylpyrazine, 2-sec-butyl-4,5-dihydrothiazole, and 2-heptanone ligands.

**Results: **According to the analysis, Rat n 1 shares 52% identity with Equ c 1, Can f 6, Fel d 4, and Mus m 1 allergens. The overlapping structures analysis  revealed high structural homology (root mean square deviation < 1). Four lineal and three discontinuous epitopes were predicted on Ra t n 1. A lineal epitope located between amino acids residues 24 and 36 was highly conserved on all allergens explored. A cross-reactive discontinuous epitope (T142, K143, D144, L145, S146, S147, D148, K152, L170, T171, T173, D174) was also found. Docking molecular simulations revealed the region involved in linking ligands, and we identified the properties of the binding of four pheromones and the binding potential of Rat n 1. Critical residues for interactions are reported in this study.

**Conclusions:** We identified some possible allergens from *Rattus norvegicus*, and those allergens could have cross-reactivity with allergens from some animals. The results need to be confirmed with *in vitro* studies and could be utilized to contribute to immunotherapy and reduce allergic diseases related to lipocalins.

## Introduction

Lipocalins are among the most important indoor/outdoor groups of animal allergens. For some, the protein structure has been resolved, but their functions are still elusive. Lipocalins generally display a low sequence identity between family members ^1^, but the lipocalin allergens are usually well preserved and can present similar patches that, in addition to serum albumins, may contribute to allergic cross-reactions among furry animals ^2– 4^. Rodents, especially mice and rats, are cosmopolitan species present in rural, periurban, and urban areas, and are most often considered as pests. In addition, the presence of these species as pets in homes and their permanent use as animal models in research laboratories have allowed constant exposure to their allergens, which is an important source of sensitization ^5, 6^.

So far, only one rat allergen has been described, Rat n 1, which is a lipocalin formed by two fractions, a prealbumin and an α-2U-globulin, secreted by the liver and found in high concentrations in urine, but also in saliva and fur ^7, 8^. Among patients allergic to rats, 87% reacted to Rat n 1 in dust ^9^. This molecule is glycosylated and, up to now, was known to have two isoforms: Rat n 1.01 (21 kDa) and Rat n 1.02 (17 kDa). Its structure is like a conventional lipocalin with eight antiparallel β chains forming a single beta sheet and an α helix to create a pocket for ligand binding, very similar to that of other allergens, such as Mus m 1, the mouse's main allergen, with a highly conserved identity ^5, 10, 11^. Four regions with potential immunodominant T cell epitopes have been described, and three of these are co-localized with the conserved regions of lipocalin, similar to the epitopes found in Bos d 2. No B cell epitopes have been reported for Rat n 1 ^12^.

Although its structure and sensitization capacity have been well described, little is known about its biological functions and how these may be related to hypersensitivity mediated by an IgE measured response and cross-reactivity with the main allergens of the most common domestic animals, dogs, cats, and horses ^13, 14^. Therefore, the objective of this study was to explore cross-reactive epitopes among Rat n 1 and homologues in domestic pets through an *in silico* approach.

## Methods

### Selection of lipocalins and alignment

The amino acid sequences of lipocalins from 5 domestic animals (Rat n 1, Mus m 1, Fel d 4, Can f 6, and Equ c 1) were selected based on the reported allergenic and phylogenetic capacity ^15^. The sequences were obtained from the UniProt database ( Table 1). Sequences that were reported by the World Health Organization (WHO)/ International Union of Immunological Societies (IUIS) Allergen Nomenclature Subcommittee and had complete sequences were used. Identity grades among lipocalins used in this study were determined by using the PRALINE web server ^16^. Parameters to perform alignment were set up to use BLOSUM62 as an exchange matrix. Three iterations were used, with an E-value of 0.01. Structural homology and root mean square deviation values were determined using UCSF Chimera (V. 1.13.1) and PDB Viewer software (v.4.10) ^17^.

**Table 1.** Allergens used in the study.

Allergen	Biological Source	Uniprot
Rat n 1	*Rattus norvegicus* (Rat)	P02761
Can f 6	*Canis familiaris* (dog)	H2B3G5
Equ c 1	*Equus caballus* (domesti chorse)	Q95182
Fel d 4	*Felis domesticus* (cat)	Q5VFH6
Mus m 1	*Mus musculus* (mouse)	P02762

### Construction of 3D model

A model of the Fel d 4 allergen was made by homology using the SWISS-MODEL server. The quality of the model was analyzed by ProSA-web. The model was refined in DeepView v.4.1 (energy minimization and rotamer replacements). Its quality was evaluated by several tools, including Ramachandran graphs, WHATIF, QMEAN4 index, and energy values (GROMOS96 force field). Three-dimensional structures of Rat n 1 (PDB:2A2G), Mus m 1 (1MUP), Can f 6 (6NRE), and Equ c 1 (1EW3) were retrieved from the Protein Data Bank.

### B epitope prediction

ElliPro and BepiPred tools were used to predict discontinuities and lineal epitopes on Rat n 1 ^18^. With ElliPro, the 3D structure of Rat n 1 was used to predict epitopes. Minimum score and maximum distance (Angstrom) were set to 0.5 and 6.

### Preparation of receptors and ligands

Preparation of receptors and ligands was carried out using the freely available Discovery Studio Visualizer 2016. Treatment of the receptors consisted of extracting the ligand and eliminating water molecules and cofactors with which their crystalline structures are resolved, followed by preparation of the ligands, making corrections in the structures, generating variations, and eliminating unwanted structures. Adding hydrogen atoms, neutralizing charged groups, generating ionization and tautomer states, obtaining alternative chiralities, and optimizing geometries were carried out.

### Docking molecular of Rat n 1 and pheromones

Using molecules identified as pheromones and the 3-dimensional molecular modeling of odorant binding protein (OBP1), docking studies were performed using SwissDock based on EADock DSS, in the following stages: (1) generation of binding modes in local and blind docking, (2) estimation of CHARMM force field energies with GRID, (3) binding of modes with the most favorable energies with FACTS and clusters, and (4) visualization of the most favorable clusters. The best-scoring docked models exhibiting the best superposition with ligands and lowest binding energy were analyzed and visualized with Chimera (V.1.13.1).

### Conservation analysis

The Rat n 1 3D structure was submitted to the ConSurf server in order to generate evolutionarily related conservation scores to help to identify functional regions in the proteins. Functional and structural critical residues in Rat n 1 sequence were confirmed by the ConSeq server.

## Results

### Rat n 1 and lipocalins exhibited identity and structural homology

**Figure 1.**
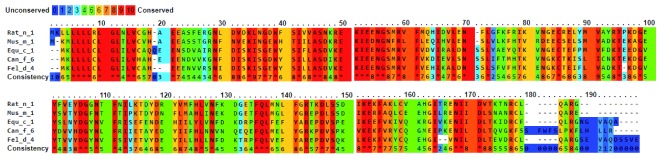
Multiple alignment among lipocalins from domestic animals. Lipocalins share a 62% of identity in their amino acid sequences.

**Figure 2.**
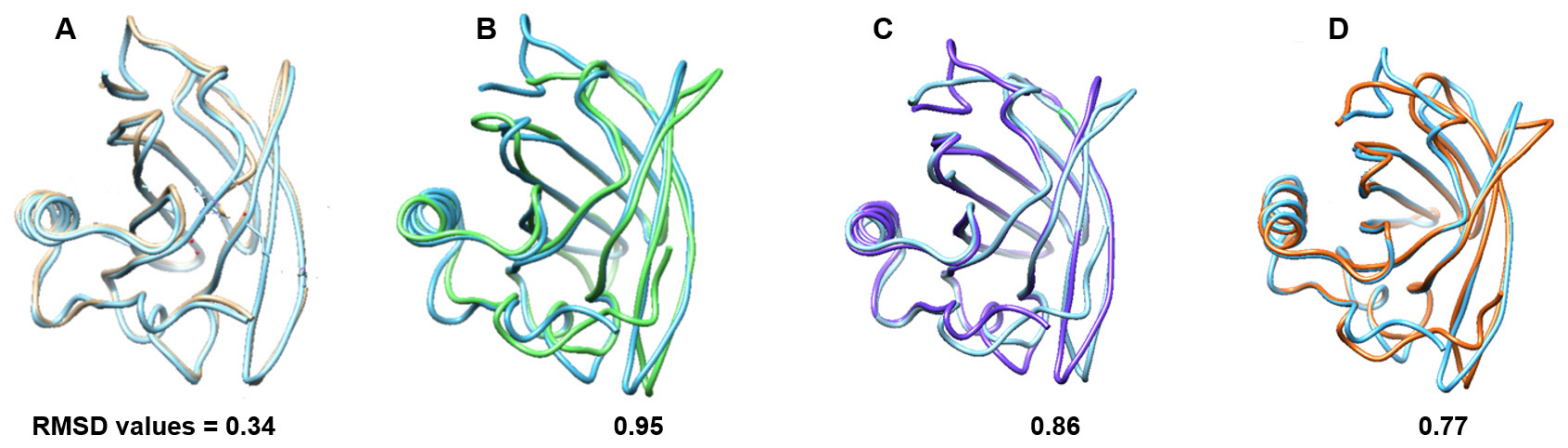
Root mean square deviation (RMSD) analysis showing structural homology among allergen Rat n 1 and homologous. ( **A**) Rat n 1 (Blue) and Mus m 1 (Brown). ( **B**) Rat n 1 and Fel d 4 (Green). ( **C**) Rat n 1 and Equ c 1 (Violet). ( **D**) Rat n 1 and Can f 6 (Orange). RMSD values are showed in Å.

Multiple alignment among amino acid sequences from Rat n 1, Can f 6, Equ c 1, Fel d 4, and Mus m 1 was performed. A 62% identity was identified among sequences compared. Residues located on positions 29 to 73 showed the highest identity. The sequence alignments of the lipocalins showed that identical residues formed short continuous segments ( Figure 1). A comparison of the secondary structural elements of Rat n 1 with the structures listed in Table 1 revealed backbone atomic RMSD values between 0.3 and 0.95 Å, with Mus m 1 showing the most closely related structure and sequence homology to Rat n 1. For all structures analyzed, the closest structural homology was found on the α-helical amino acid sequence spanning region on Rat n 1 containing nine conserved residues (IKEKFAK-L) ( Figure 2). While these proteins showed the same overall fold change, some detailed structures contained differences, such as major structural differences located on loop regions for all allergens in this study.

### Linear and discontinuous epitopes were predicted on lipocalins

**Figure 3.**
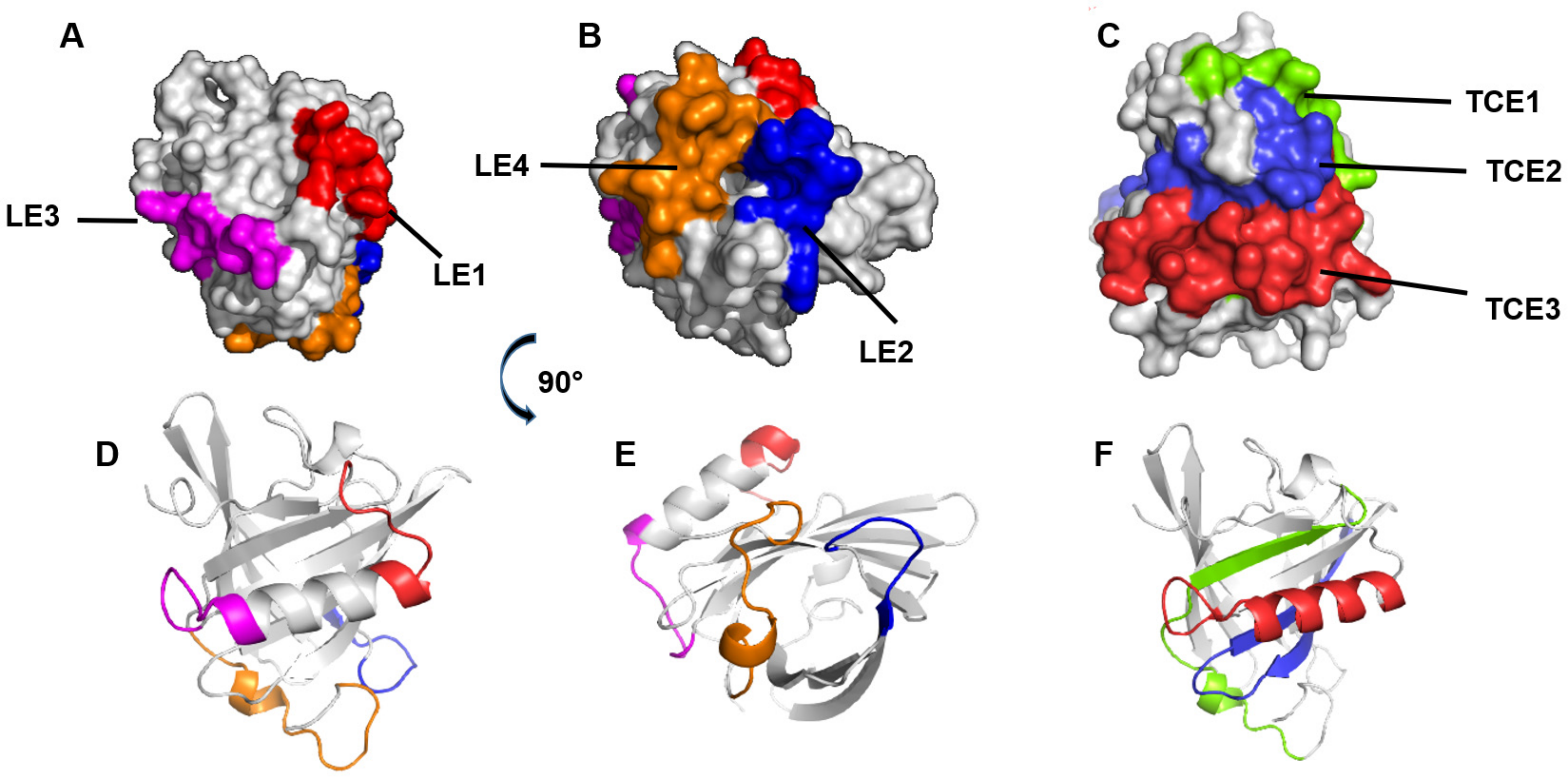
Lineal epitopes predicted on Rat n 1. ( **A**– **C**) Surface models showing areas covered by predicted epitopes 1-4. ( **D**– **F**) Cartoon models showing location of predicted epitopes on 3D structure.

Using ElliPro and BepiPred servers, four lineal and three discontinuous epitopes on Rat n 1 were predicted ( Table 2 and Table 3). The first and third epitopes were located on α-helices, spanning residues 158–165 and 141–148 ( Figure 3). Both epitopes had a surface area of 300 Å.

**Table 2.** Lineal epitopes predicted by Ellipro and Bepipred servers. LE (Lineal epitope).

Epitope	Start	End	Peptide	Number of residues	Score
LE1	158	165	EAHGITRD	8	0.715
LE2	91	97	YKTPEDG	7	0.679
LE3	141	148	RTKDLSSD	8	0.659
LE4	24	36	STRGNLDVAKLNG	13	0.608

**Table 3.** Discontinous Epitopes predicted by Ellipro. DE (Discontinous Epitope).

Epitope	Residues	Number of residues	Score
DE1	R76, K78, E79, N80, G81, E82, C83, R84, E85, C176	10	0.839
DE2	E102, Y103, D104, G105, F127, K128, N129, G130, E131, T132	10	0.755
DE3	T142, K143, D144, L145, S146, S147, D148, K152, L170, T171, T173, D174	12	0.596

**Figure 4.**
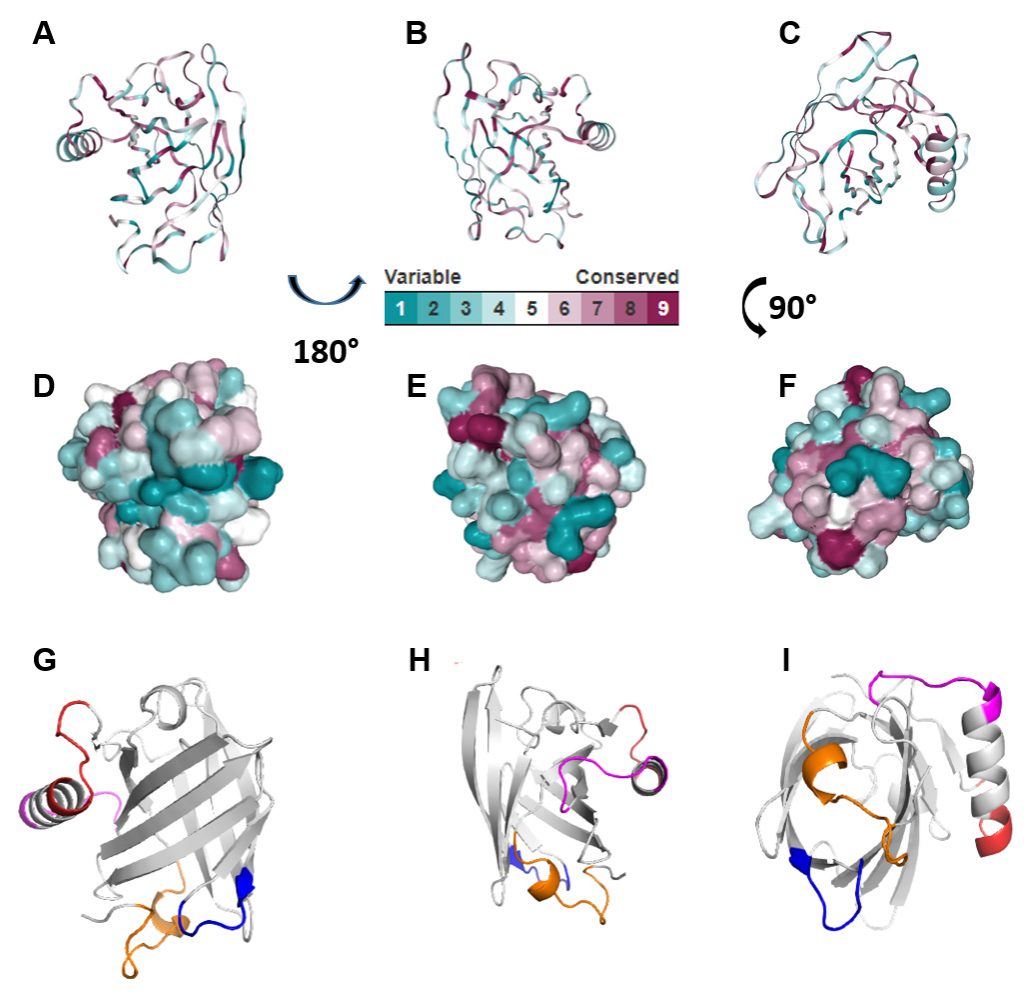
Phylogenetic analysis of lipocalins by using Consurf. ( **A**– **C**) Ribbon models showing conserved region among lipocalins. ( **D**– **F**) Surface models showing conserved region among lipocalins. ( **G**– **I**) Cartoon models showing linear epitopes predicted.

The third epitope was identified as being cross-reactive among Rat n 1, Mus m 1, Equ c 1, Can f 6, and Fel d 4. From all residues conforming with mapped epitopes, 80% were conserved and surface exposed among lipocalins analyzed in this study. The second and fourth epitopes were located on loop regions, spanning residues 91–97 and 24–36 with surface areas of 262 and 487 Å, respectively ( Figure 3). Conservative analysis indicated that both regions were highly conserved in the lipocalin family ( Figure 4). According to ConSurf analysis, the region covering the second lineal epitope is conserved among the lipocalin family.

**Figure 5.**
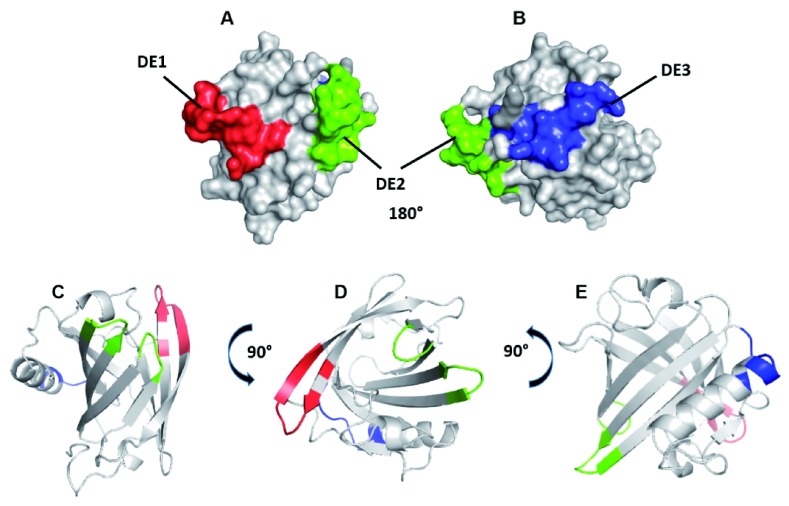
( **A**, **B**) Surface models showing “discontinuous epitopes predicted by Ellipro server. ( **C**– **E**) Cartoon models showing location of residues conforming epitopes discontinues.

The first and second discontinuous epitopes were constituted by 10 amino acid residues with a surface area of 375 Å; the first discontinuous epitope was distributed on G-H and F-E β-strands and loops connecting them,where as the third epitope was mapped to an α-helical, the same region where the first lineal epitope was located ( Figure 5). This epitope contained 12 amino acid residues, and of these, 85% were surface exposed with a surface area of 487 Å.

### Rat n 1 and homologues share residues involved in pheromone ligand binding

**Figure 6.**
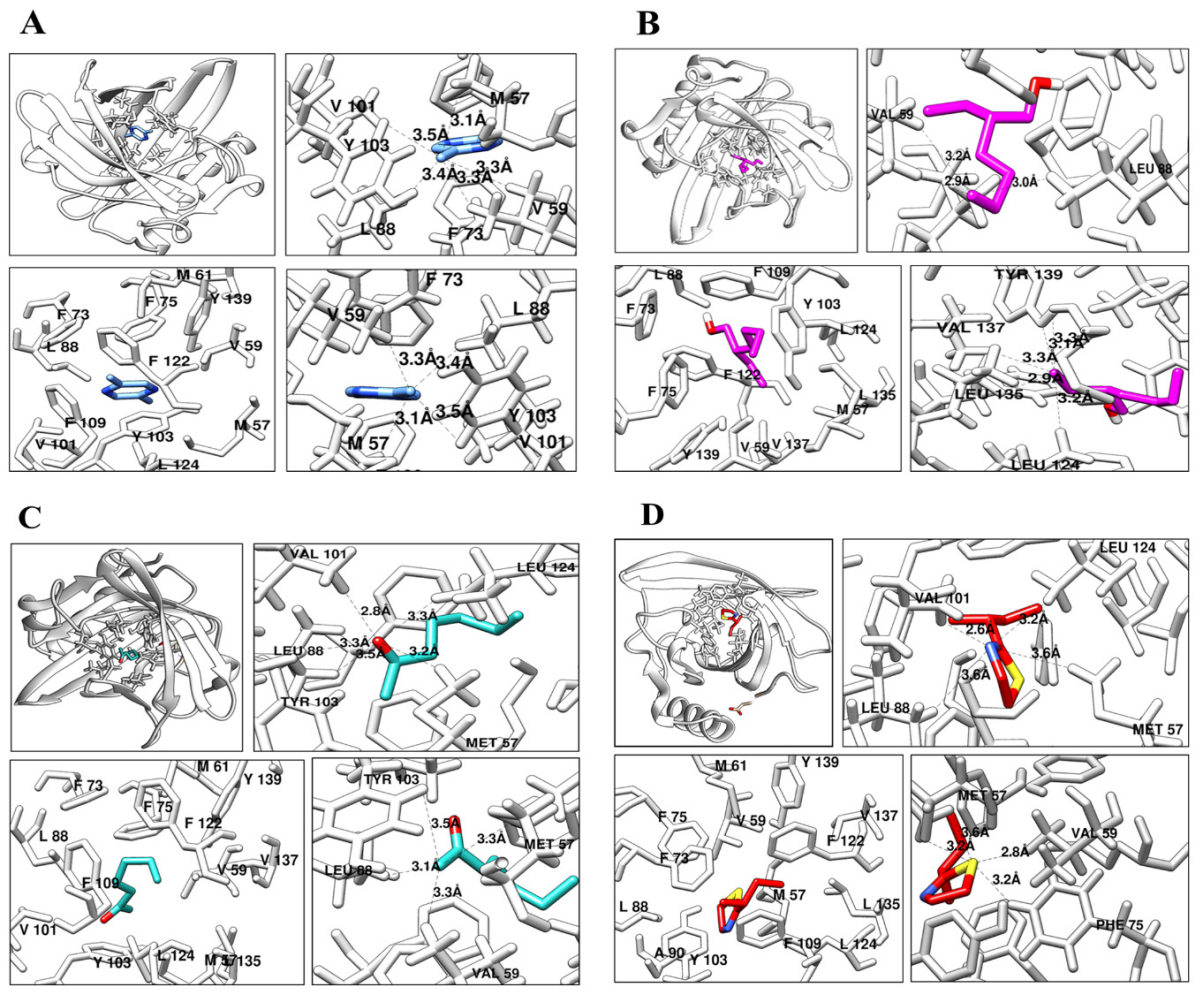
Docking analysis of pheromones – Rat n 1. A detailed view showed the docked complex with the key amino acids involved in the binding site of Rat n 1 with pheromones. ( **A**) Docked complex of Rat n 1 – 2,5-Dimethylpyrazine (DMP). ( **B**) Docked complex of Rat n 1 – 2-Ethylhexanol. ( **C**) Docked complex of Rat n 1 – 2-Heptanone. ( **D**) Docked complex of Rat n 1 – 2-sec-butyl-4,5-dihydrothiazole (SBT).

**Figure 7.**
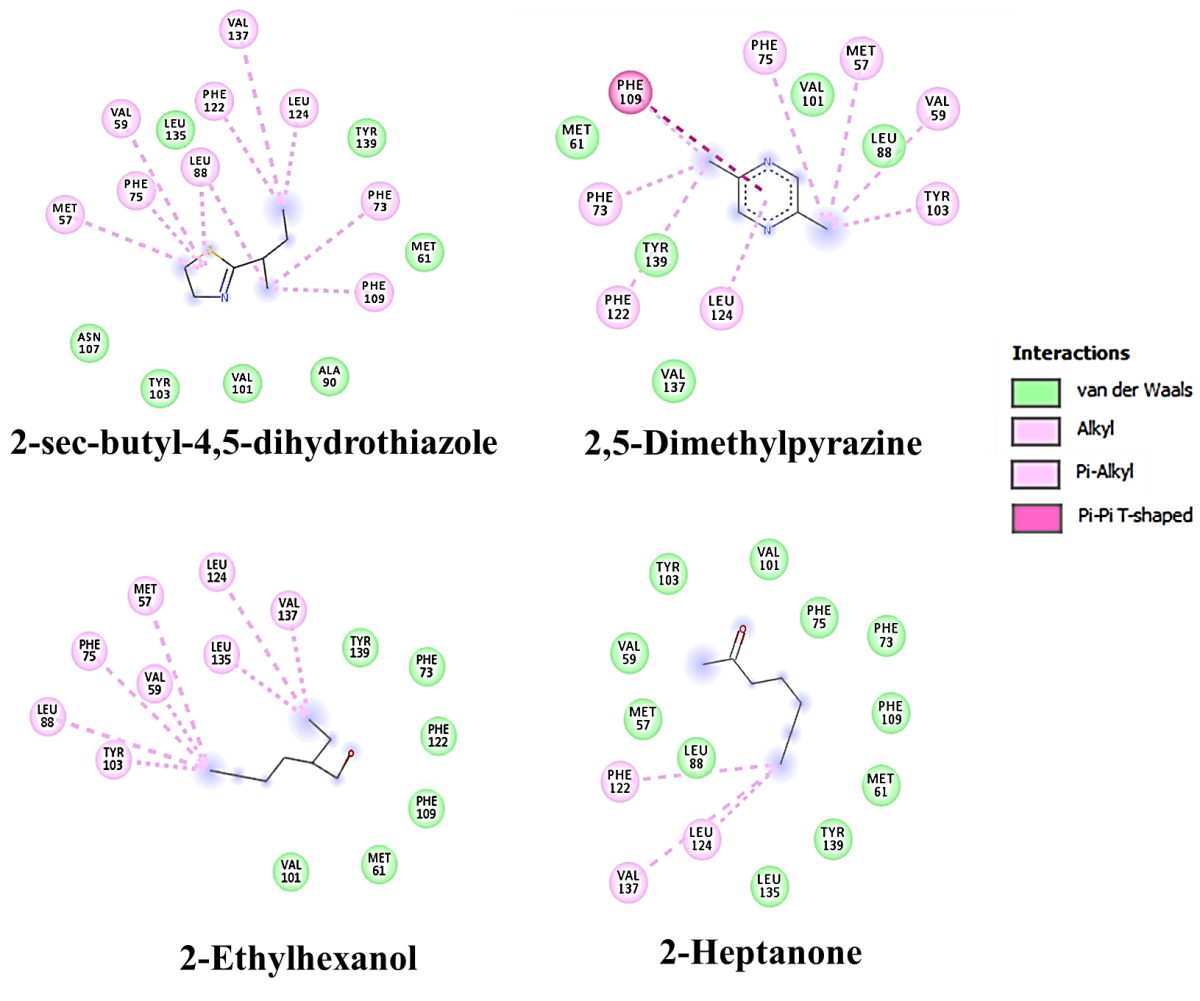
Amino acids residues of Rat n 1 and its interactions with pheromones.

Docking molecular simulations were conducted to reveal the binding site, identify the binding properties of four pheromones and the binding potential of Rat n 1. In the docked complexes ( Figure 6 and Figure 7), the central region of the corresponding structures indicated a step involving cleavage of the protein with aromatic amino acids, specifically Tyr139, Tyr103, Phe73, Phe75, and Phe122. This docked position revealed that the aliphatic structures, pyrazine derivatives, and 2-sec-butyl-4,5-dihydrothiazole (SBT) had the lowest bond energies, and the least in 2-heptanone and 2-ethylhexanol ( Table 4). Likewise, in 2,5-dimethylpyrazine the interactions of aromatic and aliphatic residues such as Met61, Leu88, Val101, Val137, and Tyr139 are described, which maintain binding to the pyrazine ring and methyl substituents in C2 and C5 ( Figure 6A–B). In the case of pheromones such as 2-ethylhexanol, a structural arrangement at the site was shown to be in contact with the aromatic and hydroxyl groups in the structure, which were shown in residues as Phe73, Phe109, Phe122 and Tyr139, not greater than 3.3 Å in angular distance ( Figure 6C). The SBT structure was in a specific orientation with the thiazoline ring in the proximal opening of the binding site. Likewise, the presence of hydrophobic interactions with apolar and aromatic residues with SBT has been established, in which alkyl-type bonds and π-alkyl are described in Met57, Val59, Leu88, and Leu124 with the thiazoline ring and structural side chain (see Figure 6D). In general, the rest of the carbon structure and the radical presentation of a structural orientation in the anterior site of the pocket in the opposite direction to the β-barrel demonstrated a relationship with the apolar residues between ethyl radicals and interactions type alkyl with Leu124, Leu135, and Val137. Similarly, 2-heptanone showed closer interaction with the protein cavity, predominantly by apolar and polar residues through hydrogen bonds, where it is common to see the relationship between the oxygen of the carbonyl group and aromatic residues of Phe75, Tyr103, and Val101; however, hydrophobic type alkylic junctions were shown with residues such as Leu124 and Val137 ( Figure 7).

**Table 4.** Binding energies for docking: molecular Rat n 1 pheromones.

Ligand	Binding	Affinity	rmsd/ub
Ratn1_2-ethylhexanol	-6.1	0.0	0.0
	-5.4	4.38	3.374
	-5.3	4.996	4.217
	-4.7	2.47	1.82
	-4.4	18.839	17.43
	-4.4	3.326	2.118
	-4.0	12.932	12.518
	-3.9	19.44	18.048
	-3.7	14.564	13.901
Ratn1_2,5-dimethylpyrazine	-5.4	0.0	0.0
	-5.4	2.646	0.025
	-5.2	2.802	1.263
	-4.9	2.62	1.205
	-4.7	2.283	1.502
	-4.7	3.461	2.219
	-4.6	2.574	1.848
	-4.1	19.67	18.453
	-4.1	19.748	18.475
Ratn1_2-sec-butyl-4,5- dihydrothiazole	-5.9	0.0	0.0
	-5.8	3.109	2.228
	-5.8	3.026	1.596
	-5.5	2.169	1.737
	-5.5	2.118	1.412
	-5.0	4.193	3.496
	-4.4	18.829	17.776
	-4.1	18.709	17.433
	-4.1	19.018	17.71
Ratn1_2-heptanone	-6.0	0.0	0.0
	-5.7	1.0	0.649
	-5.6	3445	1511
	-5.2	4094	3099
	-4.0	19139	18128
	-4.0	19475	18043
	-3.9	17091	16562
	-3.8	15193	14445
	-3.7	20058	18565

## Discussion

Animal allergens remain an important cause of sensitization and allergic diseases. Rodents such as rats are invasive cosmopolitan species that move between urban, periurban, and urban areas looking for favorable habitats and resources. The allergenicity of these species was first observed in animal caretakers and was considered an important source of occupational sensitization, affecting up to 15% of people in European countries with an active scientific community ^12^. Besides exposure in occupational settings, rodent exposure also occurs in domestic environments, as was shown in inner-city children with asthma in the USA, where rat sensitization rates were 19–21% ^19^. In contrast, a recent study from Europe reported a very low prevalence of sensitization to rodents of 0.59% in urban atopic populations without occupational exposure ^5^. Rat n 1 is the largest allergen of this species and has been well characterized. This protein belongs to the lipocalin family, a transport protein of hydrophobic ligands as lipid signaling molecules. A first approach in epitope prediction on Rat n 1 was made by Bayard *et al.* ^7^, using in silico tool Chou Fasman, however, it was impossible to determine epitopes. On the other hand, with the use of synthetic octapeptides an IgE linear epitope was mapped spanning sequence STRGNLDVAKLNG, reported in this study as LE4. Authors are clear in that discontinuous epitopes were impossible to identify using same technology.

Several major allergens are members of the lipocalin family, including those from the mouse (Mus m 1), dog (Can f 1 and Can f 2), cat (Fel d 4 and Fel d 7), horse (Equ c 1), cow (Bos d 2 and Bos d 5), hamster (Phod s 1), and rabbit (Ory c 1 and Ory c 4), among others ^3^. The factors that give rise to so many lipocalins becoming inducers of allergy are unclear. Among the allergens, cross-reactivity has been observed, mainly in organisms to which people are most exposed, such as cats, dogs, and horses.

In this study, we observed by *in silico* analysis possible crossbet-reactivity between Rat n 1, Can f 6, Equ c 1, and Fel d 4, with 62% identity among sequences compared. In previous studies, we found a high conservation state among Rat n 1, Equ c 1, and Fel d 4 (60% identity) and described possible residues with antigenic potential ^15, 20^. Nilson *et al.* ^21^ found cross-reactivity between Can f 6, Fel d 4, and Equ c 1 by inhibition assays, especially in residues located on positions 29 to 73, which showed the highest identity. In these positions, Rat n 1 also presented the highest identity with the other three lipocalins, and here we found a possible lineal epitope (LE4: Start 24 – End 36) that could explain the cross-reactivity. Jeal *et al.* ^12^ studied a population of individuals exposed to laboratory rats to determinate the proliferative response of peripheral blood mononuclear cells to Rat n 1. They found four regions as possible immunodominant T cell epitopes, and three of them were localized within the conserved regions of the lipocalins. One was also found in our study as a possible lineal epitope (LE2: Start 91 – End 97), with a high identity with Mus m 1, Equ c 1, Can f 6, and Fel d 4. The homologous allergens may contribute to multisensitization and symptoms in individuals allergic to different animals. Also, cross-reactivity to T cell epitope was found between Can f 1 and human tear lipocalin ^22^. This could support the autosensitization and increased inflammatory response mediated by T lymphocyte CD4+. Also, first T cell epitope predicted in this study shares identity with Bos d 2, a major allergen from cow ^23^. This can explain cross reactivity among rat and others allergenic sources, such as: Can f 1 and Equ c 1, which has been related to share identity to T epitope level ^24, 25^. However, Bos d 2 has been characterized as a weak inducer of immunological response ^26^.

The docking simulations demonstrated that the Rat n 1 cleft is big enough to accommodate the whole fatty acid molecule. Determining the capacity to bind some ligands by allergens is critical to understand their allergenic capacity. For Bet v 1, an allergen with capacity to link hydrophobic ligands, it has been determined that ligands such as lipids, iron, and calcium modulate its allergenicity capacity ^27, 28^. When Bet v 1 is properly loaded with iron, it can promote Th2 response. A similar property is reported for Pru p 3, a peach lipid transfer protein. Results reveal that the ligand is recognized by a type of cellular receptor called CD1d in the cell surface where the antigens appear, that is, substances able to provoke an immune system response to produce antibodies. CD1d is responsible for presenting lipid antigen to activating cells of the immune system called invariant natural killer T (iNKT) cells. Once activated, these iNKT cells produce substances that cause the characteristic symptoms of allergic disorders ^29^. Since many allergens transport diverse compounds, the discovery of Pru p 3 lipid-ligand as an adjuvant to promote allergic sensitization through its recognition by CD1d expression opened new horizons ^29^.

Of the lipocalin family, also, Mus m 1 has been characterized for pheromone linking. Experimental assays revealed that 2-sec-butyl-4,5-dihydrothiazole (SBT), 6-hydroxy-6-methyl-3-heptanone (HMH), and (±)-dehydro-exo-brevicomin (DHB) are ligands for Mus m 1. This was a first step in determining ligands with allergenic capacity ^30^. Also, some ligands could influence the stabilization of IgE conformational epitopes ^27, 31^. Here, we predicted three. So, experimental assays are needed to determine the impact of ligands in Rat n 1 on inducing allergic responses. Resolution of 3D structure of Rat n 1 by X-ray, it helped to understand structural basis for linking ligands. According to Bocskei *et al.* ^32^, residues Tyr24, Val58, Ala107 and Phe94, are critical to lipocalin activity. However, none of these residues was identified in docking assay.

For other allergens, such as Fel d 1, lipid ligands enhance TLR4 activation and innate immune signaling and promote airway hypersensitivity reactions in diseases such as asthma ^33^. For Bla g 4, a lipocalin from Blattella germanica (German cockroach), a capacity to bind hydrophobic ligands such as: tyramine and octopamine has been characterized ^34^. But residues involved in linking ligands in Rat n 1 are not conserved in Bla g 4, this is relevant because suggest a capacity to link different kinds of ligands in both allergens.

The outcomes of the current work include (1) a comprehensive understanding of the structure of Rat n 1 protein and structural similarities and differences between Rat n 1 and other lipocalins, and (2) a structural and molecular basis for the identification of epitopes responsible for cross-allergenicity between rat and domestic animal allergenic lipocalins. These epitopes may contribute significantly to designing rational strategies for diagnosis of and immunotherapy for domestic animal allergies.

## Data availability

All data underlying the results are available as part of the article and no additional source data are required.
