# Supplementary material for: In silico analysis of a major allergen from Rattus norvegicus, Rat n 1, and cross-reactivity with domestic pets
Source: F1000Res. 2019 Dec 12;8:1707. Originally published 2019 Oct 1. [Version 2] doi: 10.12688/f1000research.20534.2 (PMC7194344; doi:10.12688/f1000research.20534.2)
Supplement: Peer review history

# Peer review: Reviewer response for version 2

**DOI:** 10.5256/f1000research.23935.r60848

**Published:** 2020-04-14

**Authors:** Enrique Fernandez-Caldas

**Affiliations:** 1 Inmunotek SL, Madrid, Spain; 2 College of Medicine, University of South Florida, Tampa, FL, USA

**General Comments:**

- Lipocalins play a role in the cellular trafficking of pheromones and are involved in allergic responses to domestic pets.
- Cross-reactivity among allergens of this group has been poorly explored, and the pheromone linking capacity is not well characterized.
- The Main objective of this study was to investigate cross-reactive epitopes and pheromone linking capacity among Rat n 1, and homologues in domestic pets, through an in silico approach.
- The ElliPro and BepiPred in silico tools were used to predict B cell linear and cross-reactive epitopes. The pheromone linking capacity was explored by docking virtual screening with 2-ethylhexanol, 2,5-dimethylpyrazine, 2-sec-butyl-4,5-dihydrothiazole, and 2-heptanone ligands.
- Rat n 1 shares 52% identity with Equ c 1, Can f 6, Fel d 4, and Mus m 1 allergens. The analysis of the overlapping structures revealed high structural homology. Four lineal and 3 discontinuous epitopes were predicted on Ra t n 1. A lineal epitope located between amino acids residues 24 and 36 was highly conserved on all allergens explored. A cross-reactive discontinuous epitope was also found. Docking molecular simulations revealed the region involved in linking ligands. The authors identified the properties of the binding of four pheromones and the binding potential of Rat n 1.
- The authors Critical residues for interactions are reported in this study.
- The authors have identified some possible allergens from *Rattus norvegicus*, and those allergens could have cross-reactivity with allergens from other mammalian species.  The results should be confirmed in in vitro studies and could be utilized to design specific immunotherapy products to treat allergic diseases related to lipocalins.

 

**Specific Comments:**

- This is a very interesting well-presented study. The Introduction contains the relevant information and in the Discussion section, the results are well compared and discussed.
- The article may contain too many figures and Tables, and some of them could be replaced with a complete description in the text and avoid duplication.
- The abstract has been revised by this reviewer and could be interesting for the authors to see my suggestions/corrections.
- Some of the conclusions may be too far reaching and could be changed.

Is the work clearly and accurately presented and does it cite the current literature?

Yes

If applicable, is the statistical analysis and its interpretation appropriate?

Yes

Are all the source data underlying the results available to ensure full reproducibility?

Yes

Is the study design appropriate and is the work technically sound?

Yes

Are the conclusions drawn adequately supported by the results?

Yes

Are sufficient details of methods and analysis provided to allow replication by others?

Yes

Reviewer Expertise:

Allergen and allergen immunotherapy.

I confirm that I have read this submission and believe that I have an appropriate level of expertise to confirm that it is of an acceptable scientific standard.

# Peer review: Reviewer response for version 2

**DOI:** 10.5256/f1000research.23935.r57804

**Published:** 2020-01-08

**Authors:** Anna Pomés

**Affiliations:** 1 Indoor Biotechnologies Inc., Charlottesville, VA, USA

The authors seem to have responded to most of my comments, but few, mostly minor, still need to be addressed:

- “Line 23-24 of introduction: The reference Böcskei et al. Nature 1992 should be added to the explanation of the structure of Rat n 1. Similarly, under methods section: “Construction of 3D models”, the pdb codes of the structures should be provided.”
- In abstract: Rat n 1 shares 52%. Is this correct, or should it be 62% as mentioned later in the paper? The percentage of identity should be corrected if needed for consistency.

**Minor comments:**

 In the paragraph before last: “Blattella germanica (German cockroach), a capacity to bind hydrophobic ligands such as: tyramine and octopamine has been characterized34. But residues involved in linking ligands in Rat n 1 are not conserved in Bla g 4, this is relevant because suggest a capacity to link different kinds of ligands in both allergens.”

- *Blattella germanica *should be in italics. 
- “This is relevant because suggest a capacity to link different kinds of ligands in both allergens.” Should be a separate sentence.

In Table 1:

- *Equus caballus* (domesti chorse) should read: domestic horse

Before last paragraph in discussion:

- “Resolution of 3D structure of Rat n 1 by X-ray, it helped to understand structural basis for linking ligands”. This sentence should be: Resolution of 3D structure of Rat n 1 by X-ray helped to understand structural basis for binding ligands.

Is the work clearly and accurately presented and does it cite the current literature?

Partly

If applicable, is the statistical analysis and its interpretation appropriate?

Not applicable

Are all the source data underlying the results available to ensure full reproducibility?

Yes

Is the study design appropriate and is the work technically sound?

Partly

Are the conclusions drawn adequately supported by the results?

No

Are sufficient details of methods and analysis provided to allow replication by others?

Yes

Reviewer Expertise:

Allergen characterization and epitope mapping.

I confirm that I have read this submission and believe that I have an appropriate level of expertise to confirm that it is of an acceptable scientific standard.

# Peer review: Reviewer response for version 1

**DOI:** 10.5256/f1000research.22569.r54531

**Published:** 2019-10-14

**Authors:** Anna Pomés

**Affiliations:** 1 Indoor Biotechnologies Inc., Charlottesville, VA, USA

The manuscript by Munera et al. performed an *in silico* analysis of the major rat allergen Rat n 1, and explored cross-reactive epitopes and pheromone linking capacity among Rat n 1 and homologous allergens from domestic pets. The analysis is interesting from a molecular-structural point of view, but the paper needs to be put in context in the allergy field, including previous literature.

1. The structures of Rat n 1 and Mus m 1 were published in Nature in 1992 and reported “the role of these proteins in pheromone transport” and elaborated on “the structural basis of ligand binding”. This publication should be cited, and the information about the ligand binding taken into consideration for the analysis performed. For example, how the ligands analyzed compare to the pheromones reported in the paper would be very interesting. This is the reference:
  Böcskei Z, Groom CR, Flower DR, Wright CE, Phillips SEV, Cavaggioni A, Findlay JBC, North ACT. Pheromone binding to two rodent urinary proteins revealed by X-ray crystallography. Nature 1992; 360:186–188. ^1^
  Therefore, the sentence in page 9: “Of the lipocalin family, only Mus m 1 has been characterized for pheromone linking” needs correction.
2. Another lipocalin whose ligand was identified is cockroach Bla g 4, and this study should be cited and the ligand analyzed and compared with the ones used in this study for a comment in the discussion. Ref: The major cockroach allergen Bla g 4 binds tyramine and octopamine. Offermann LR, Chan SL, Osinski T, Tan YW, Chew FT, Sivaraman J, Mok YK, Minor W, Chruszcz M. Mol Immunol. 2014 Jul;60(1):86-94. ^2^
3. Mapping of B and T cell epitopes has been performed before for some lipocalins (i.e. Dr. Virtanen’s group in Finland). It would be good if the in silico analysis could be related to experimentally identified epitopes, and to discuss the results in the context of previous studies, such as:
  Bayard C, Siddique AB, Berzins K, Troye-Blomberg M, Hellman U, Vesterberg O. Mapping of IgE binding regions in the major rat urinary protein, alpha 2u-globulin, using overlapping peptides. Immunol Invest. 1999 Sep-Dec;28(5-6):323-38. ^3^
  Zeiler T, Mäntyjärvi R, Rautiainen J, Rytkönen-Nissinen M, Vilja P, Taivainen A, Kauppinen J, Virtanen T. T cell epitopes of a lipocalin allergen colocalize with the conserved regions of the molecule. J Immunol. 1999 Feb 1;162(3):1415-22. ^4^
  Kinnunen T, Buhot C, Närvänen A, Rytkönen-Nissinen M, Saarelainen S, Pouvelle-Moratille S, Rautiainen J, Taivainen A, Maillère B, Mäntyjärvi R, Virtanen T. The immunodominant epitope of lipocalin allergen Bos d 2 is suboptimal for human T cells. Eur J Immunol. 2003 Jun;33(6):1717-26. ^5^
  Immonen A, Farci S, Taivainen A, Partanen J, Pouvelle-Moratille S, Närvänen A, Kinnunen T, Saarelainen S, Rytkönen-Nissinen M, Maillere B, Virtanen T. T cell epitope-containing peptides of the major dog allergen Can f 1 as candidates for allergen immunotherapy. J Immunol. 2005 Sep 15;175(6):3614-20. ^6^
  Immonen A, Kinnunen T, Sirven P, Taivainen A, Houitte D, Peräsaari J, Närvänen A, Saarelainen S, Rytkönen-Nissinen M, Maillere B, Virtanen T. The major horse allergen Equ c 1 contains one immunodominant region of T cell epitopes. Clin Exp Allergy. 2007 Jun;37(6):939-47. ^7^
4. The rationale for selecting Can f 6 for the study should be explained since there are other dog lipocalins (Can f 1, Can f 2, Can f 4).
5. Abstract-results:
  a) “The overlapping structures assay”. The use of “analysis” instead of “assay” is best.
  b) “docking …revealed the active site”. The expression “active site” is especially used for enzymes, and for binding without catalytic activity involved the term “binding site” might be more appropriate. The authors need to correct where it applies (e.g. page 5, line 21).
  c) If Rat n 1 shares 52% amino acid identity with each of the four allergens, then the sentence is correct, but this might need revision if the percentages are different for each animal.  In contrast, in Results, line 6, and in the discussion (third paragraph) 62% identity is reported. The authors should revise this inconsistency. Discussion, line 4 of third paragraph: “high percentage of identity” is better than “high conservation state”.
6. In the introduction, line 20: “Among patients allergic to rat, 73% and 87% reacted to Rat n 1 in dust”. Not clear why there are two percentages in this sentence.  Is some information missing?
7. Line 23-24: The reference above Böcskei et al. Nature 1992 should be added to the explanation of the structure of Rat n 1. Similarly, under methods section: “Construction of 3D models”, the pdb codes of the structures should be provided.
8. In Table 3: The abbreviations are not clear: If LE is discontinuous epitope (table title), then why in Figure 3 LE are linear epitopes? Are TCE, T-cell epitopes?  Please define abbreviations.
9. The proper allergen nomenclature should be used to name lipocalins from hamster: Phod s 1, Mes a 1 (instead of Pho 21kD) in page 8 (discussion, second paragraph). Nomenclature can be found in the official WHO/IUIS Allergen Nomenclature database ( www.allergen.org).
10. Bet v 1 is not a lipocalin family member as indicated in paragraph 4 of the discussion. This needs to be corrected.

Minor comments:

- Table 1: species names should be in italics
- Page 5, line 11, “…constituted by 10 amino acid residues…”
- Legend to Figure 5: “discontinuous epitopes” instead of “epitopes discontinues”.
- Discussion: second paragraph: “lipocalin family” instead of “lipocal infamily”.
- Paragraph 4 of discussion: use of “enormous” should be avoided.
- Before last paragraph of discussion: “This is the first exploration of the capacity of Rat n 1 to bind pheromones”. This is an in silico assay, and the study mentioned above (Böcskei et al) proved pheromone binding by X-ray crystallography.

Is the work clearly and accurately presented and does it cite the current literature?

Partly

If applicable, is the statistical analysis and its interpretation appropriate?

Not applicable

Are all the source data underlying the results available to ensure full reproducibility?

Yes

Is the study design appropriate and is the work technically sound?

Partly

Are the conclusions drawn adequately supported by the results?

No

Are sufficient details of methods and analysis provided to allow replication by others?

Yes

Reviewer Expertise:

Allergen characterization and epitope mapping.

I confirm that I have read this submission and believe that I have an appropriate level of expertise to confirm that it is of an acceptable scientific standard, however I have significant reservations, as outlined above.

## References

[1] : Pheromone binding to two rodent urinary proteins revealed by X-ray crystallography. *Nature*.1992;360(6400) : 10.1038/360186a0 186-8 10.1038/360186a0 1279439

[2] : The major cockroach allergen Bla g 4 binds tyramine and octopamine. *Mol Immunol*.2014;60(1) : 10.1016/j.molimm.2014.03.016 86-94 10.1016/j.molimm.2014.03.016 24769496PMC4097008

[3] : Mapping of IgE binding regions in the major rat urinary protein, alpha 2u-globulin, using overlapping peptides. *Immunol Invest*.28(5-6) :323-38 1057463010.3109/08820139909062266

[4] : T cell epitopes of a lipocalin allergen colocalize with the conserved regions of the molecule. *J Immunol*.1999;162(3) :1415-22 9973397

[5] : The immunodominant epitope of lipocalin allergen Bos d 2 is suboptimal for human T cells. *Eur J Immunol*.2003;33(6) : 10.1002/eji.200322952 1717-26 10.1002/eji.200322952 12778490

[6] : T cell epitope-containing peptides of the major dog allergen Can f 1 as candidates for allergen immunotherapy. *J Immunol*.2005;175(6) : 10.4049/jimmunol.175.6.3614 3614-20 10.4049/jimmunol.175.6.3614 16148105

[7] : The major horse allergen Equ c 1 contains one immunodominant region of T cell epitopes. *Clin Exp Allergy*.2007;37(6) : 10.1111/j.1365-2222.2007.02722.x 939-47 10.1111/j.1365-2222.2007.02722.x 17517108

# Response

**Published:** 2019-11-27

**Authors:** Marlon Munera

Dear, 

Reviewer. 

We have taken note of all your comments. 

1.  The sentence in page 9: “Of the lipocalin family, only Mus m 1 has been characterized for pheromone linking” needs correction.

R. Correction was made. 

2. Ref: The major cockroach allergen Bla g 4 binds tyramine and octopamine. Offermann LR, Chan SL, Osinski T, Tan YW, Chew FT, Sivaraman J, Mok YK, Minor W, Chruszcz M. Mol Immunol. 2014 Jul;60(1):86-94

R. Reference suggested was added.

3. It would be good if the in silico analysis could be related to experimentally identified epitopes, and to discuss the results in the context of previous studies.

R. All references suggested were incorporated 

4. The rationale for selecting Can f 6 for the study should be explained since there are other dog lipocalins (Can f 1, Can f 2, Can f 4).

R. Can f 6 was selected because share a moderated identity level with Rat n 1 and its 3D structure is resolved experimentally. 

5-10. Abstract-results

All grammar advice and corrections were incorporated. 

On behalf of all authors, we appreciate all your comments to improve our research. 

.
